# Rapid shift in greenhouse forcing of emerging arctic peatlands

**DOI:** 10.1038/s41598-023-29859-4

**Published:** 2023-02-17

**Authors:** Torben R. Christensen, Johan Scheller, Maria Scheel, Daniel Alexander Rudd, Marcin Jackowicz-Korczynski, Mikhail Mastepanov, Efrén López-Blanco

**Affiliations:** 1 Department of Ecoscience, Aarhus University, Roskilde, Denmark; 2 Oulanka Research Station, Oulu University, Kuusamo, Finland; 3 Greenland Institute for Natural Resources, Nuuk, Greenland

**Keywords:** Biogeochemistry, Carbon cycle, Climate change

## Abstract

In this study, we hypothesised that the actual development stage (i.e., current age of the ecosystem) is a determining factor for the magnitude of methane production and emissions in young, northern high-latitude peatlands. We demonstrate that the earliest development of peat soil imposes a sink-to-source shift in the greenhouse warming potential of emerging peatlands in response to climate change that holds feedback mechanisms of importance for short-term (< 100 years) climate warming.

## Introduction

The northern high latitudes represent a substantial atmospheric source of methane emissions and will be subject to temperature and precipitation increases with projected climate change^1^. Northern peatlands are a key contributor to this Arctic atmospheric methane source, and it is pivotal to understand the spatial and temporal factors controlling the observed spatial variability in their emissions. Microclimate and plant species composition are well-known drivers that influence the scale of net emissions^2^. The storage of soil organic carbon (SOC) in cold, waterlogged peatlands is especially facilitated by low microbial decomposition rates in relation to the input of plant organic matter^3^. The response of the microbial metabolism to increasing temperature is highly variable and difficult to monitor.

We investigated multiple datasets from a peatland located in the Zackenberg valley, NE Greenland^4^, which has been subject to a large number of previous methane flux studies^4^ and a wide range of related experiments and process-based modelling efforts^5–7^. We focus on the particular hypothesis that the peat age and peatland development stage strongly influence the spatial variability in methane fluxes, and we use radiocarbon dating of the basal peat age in distributed locations of the fen to study this. If the hypothesis is true, this will in turn add additional explanatory power to ecosystem models representing the spatial distribution of methane emissions.

## Results and discussion

Despite different focusses in previous methane flux studies from Zackenberg, they all include comparable control measurements in undisturbed plots^4^. When comparing these studies, the fluxes vary by a factor of more than five over relatively short distances (less than 100 m). Although part of this variability can be explained by interannual fluctuations, a substantial variation in base fluxes remains when the temporal aspect is excluded. Base flux is here defined as the flux measured in undisturbed (by experimental treatments, i.e., control plots) locations of our studied fen peatland on comparable dates (July and August only) and times of day and obtained using similar flux chamber methodologies. An overview of these different methodologies is provided in Scheller et al.^4^.

**Figure 1:**
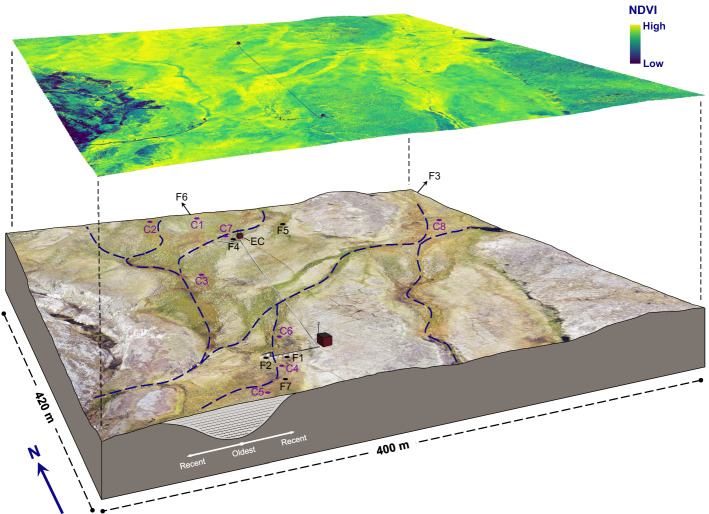
Angled view of the DEM-based orthophoto illustrating the Zackenberg fen site across a 400 m × 450 m area. The map includes the locations of all cores (Table 1a), CH_4_ flux study sites (Table 1b) and EC tower (Table S2) together with an indication of the main surface water flow direction. Additionally, an NDVI map (August 2020) characterising the local plant greenness overlays the map. The areas surrounding the main flow are found to hold the older, deeper peat deposits and hence higher methane emissions. In support of this, the older peat age determinations at C3, C6, C7, and C8 are all located centrally to the hydrological flow lines and coincide with the vicinity of high mcrA gene counts (for C3) and fluxes measured. F3 and F6 are both located approximately 300 m from the edge of the map. Note that the surface topography, infrastructure, and subsurface processes are not to scale. Image source: Greenland Ecosystem Monitoring, 2020.

In addition to direct physical drivers such as temperature and soil moisture, vascular plant species composition and density are known to act as drivers/controls of base fluxes^2^. Detailed information from multispectral analyses of the vascular plant cover (and possibly species-level recognition) may be used for extrapolation purposes that relate to the part of the methane flux variability associated with this factor (Fig. 1). However, the substantial variability observed from multiple flux studies summarized in Fig. 1 and Table 1 comes from periods with similar microclimatic conditions and locations sharing a homogeneous vegetation cover (dominated by *Dupontia psilosantha* and *Eriophorum scheuchzeri* wet tundra peatland). This suggests that other factors, such as the soil/peat development stage, may also contribute to explaining the documented flux variability.

**Table 1:** Parameter tables for (a) location analysis including basal peat ^14^C age and mcrA gene counts and (b) location of multiple years of comparable flux study sites.

(a)				
Site	^14^C age (BP and fM)	Age (year AC)	mcrA (fg/µl)	References
C1	100 ± 30	1850	3.80 ± 1.87	This study
C2	1.492 ± 0.0006 fM	(1972)	4.05 ± 1.21	This study
C3	180 ± 30	1770	19.74 ± 4.61	This study
C4	60 ± 35	1890	0.96 ± 0.12	This study
C5	210 ± 30	1740	7.75 ± 3.54	This study
C6	375 ± 30	1575	–	Palmtag et al. (2015)^10^
C7	1170 ± 35	780	–	Palmtag et al. (2015)^10^
C8	840 ± 30	1110	–	Palmtag et al. (2015)^10^

(b)				
Site	Studies	Study years	Mean flux (mg CH_4_ m^–2^ h^–1^)	Reference(s)
F1	1	14	1.3	Mastepanov et al. (2013^9^; in prep.)
F2	1	8	3.2	Mastepanov et al. (2013^9^; in prep.)
F3	1	3	3.4	Ström et al. (2015)^6^
F4	2	5	5.8	Ström et al. (2012)^11^; Falk et al. (2014)^12^
F5	1	1	6.8	Tagesson et al. (2013)^13^
F6	1	1	6.9	Christensen et al. (2000)^14^
F7	1	2	7.4	Joabsson and Christensen (2001)^15^

Site codes refer to Fig. 1. Radiocarbon ages are given in years in BP (before present = AD 1950) from 1650 to 1950 and in fM (fraction modern) for samples younger than 1963 (post-weapons testing).

The spatial distribution (Fig. 1) and magnitude variability (Table 1b) of 12 published studies focusing on various aspects of methane fluxes in the Zackenberg fen peatland area over the time period 1997–2021 was previously investigated^4^. The base fluxes reported by all these studies characterising the fen peatland area span a range from < 1 to > 8 mg CH_4_ m^−2^ h^−1^. Such an extensive range suggests that multiple processes are involved, and there is a continued need to better understand the underlying mechanisms controlling this peatland spatial variability.

To examine the flux variability in more detail, we collected soil cores randomly from the fen peatland area and analysed them for ^14^C age and methanogenic activity (Table 1a and Table S1). Additional earlier published ^14^C age data from the same fen area were also included. This particular fen peatland reveals recent ecosystem development dating back approximately one thousand years and with large areas only emerging as peatland ecosystems (vegetated wet tundra as opposed to barren or floodplain) since the Little Ice Age (fourteenth to eighteenth century). As a result, this cold period has left a very short time for actual peat development. Most northern peatlands date back much further in time (averaging 4–5 k years^8^) and include deeper peat deposits. However, current growing season methane emissions in several in parts of the Zackenberg fen are already comparable in strength to the emissions from deeper peatlands in southern parts of the Arctic^9^.

This recent development of the studied tundra landscape may resemble the general future for the entire Arctic domain as a consequence of predicted climate warming^16^. These consequences also include further permafrost thawing and subsequent changes in productivity and peat formation^16^.

The fen peatland in Zackenberg has been reported as a CO_2_ sink in all years except one (2018) associated with a substantial late snowmelt period (due to an anomalously snow-rich winter) as measured over more than 10 years using the eddy covariance (EC) technique^7^ (Fig. 1 and Table S2). It is further expected that the CO_2_ sink strength of this fen system will continue to increase, with an additional 10 g CO_2_ m^−2^ year^−1^ by the end of 2100^17^. The central part of the fen (Location 74.48°N, −20.56°E) is now also a certified Class 2 station in the Integrated Carbon Observing System (ICOS), and 2021 included the first-ever complete winter season (including the November–April period during which Zackenberg Research Station is unattended). The annual C uptake is higher than that in another more southern tundra fen site in SE Greenland due to its documented nutrient-richer conditions^7^. The annual CO_2_ uptake varies between −90 g CO_2_ m^−2^ year^−1^ and + 21 g CO_2_ m^−2^ year^−1^, with an average of −50 g CO_2_ m^−2^ year^−1^ ^7^ over a decadal scale of measurements (Table S2). Therefore, the consideration of methane as a key component of the greenhouse gas (GHG) balance of this fen peatland ecosystem needs to be aligned with the significant variability in the CO_2_ sink functioning.

We hypothesised that the early stages of organic soil and peat formation and development could initiate a rapid increase in methane emissions relative to a barren landscape. The spatial difference among sites related to basal peat age could then also be a factor determining the observed local-scale differences in base fluxes.

Microbial methane cycling includes methane-producing, methanogenic, methane-releasing, and methanotrophic processes, which have been studied in peatlands before^18^. While methane oxidation can offset peatland methane emissions^3^, we investigated whether the abundance of methyl-coenzyme M reductase (mcrA), a key methanogenesis metabolism gene, as a proxy for the abundance of methanogenic activity^19^, increases with the onset of early organic layer/peat development. The highest mcrA gene counts were found to coincide with the oldest BP age and highest methane emissions, although no statistical correlation could be found.

Relatively older samples are situated close to the main hydrological flow lines crossing the fen (Fig. 1) and are associated with the presence of methane released from vascular plants but also the presence of peat-forming mosses in the ground layer that in turn stimulate the initiation of peat development. All these factors combined may then lead to a spatial pattern associated with higher methanogenesis gene counts in the older parts of the peat (Table 1a). This may potentially further contribute to the spatial pattern of genes that suggest/align with a spatial pattern of overall methane fluxes (Table 1b). The above-described independent lines of evidence led us to assume that these flow lines were the likely initiation centres for the onset of fen peatland development, which would fit with early and more recent studies on early formation patterns^20,21^.

The combined in situ observations (Table 1) provide the possibility of evaluating the total GHG balance associated with the early development stages over a few hundred years of an emerging the peatland ecosystem in the high Arctic. This process also suggests the potential development towards conditions already prevailing at lower latitudes. The observed and predicted trends towards warmer and wetter conditions in the Arctic will lead to more widespread wet tundra peatland development associated with the “greening” of the tundra^22^. The development of the GHG balance of these changing landscapes will, therefore, also hold important implications for further climate development and hence warrant inclusion in Earth system models^23^.

**Figure 2:**
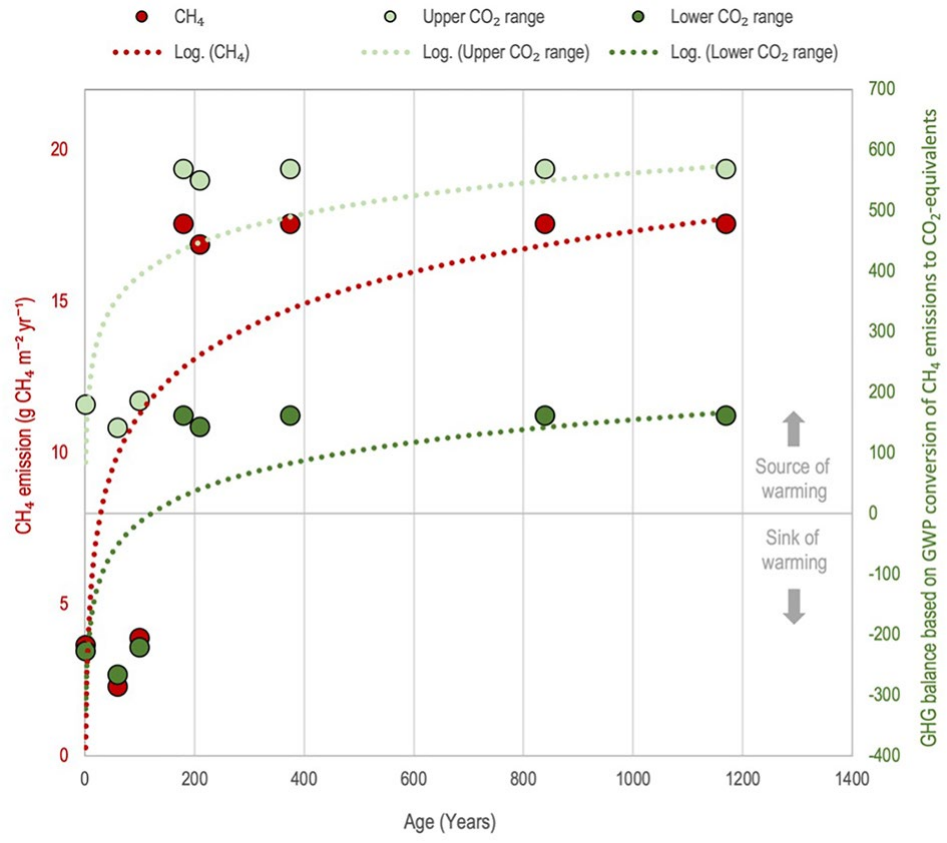
Annual methane emissions plotted against basal peat ages from closest vicinity locations (red dots, left axis). Furthermore, the GHG balance was calculated using a standard global warming potential (GWP) calculation (100 year GWP CH_4_: 28) for methane in CO_2_ equivalents combined with the annual fen CO_2_ flux range from the 11-year eddy covariance dataset (upper + 23 g CO_2_-C m^−2^ year^−1^, lower – 90 g CO_2_-C m^−2^ year^−1^, respectively; Table S2) (light and dark green dots, right axis). Note the age range (0–100 years) depending on CO_2_ sink strength where the fen ecosystem shifts from being a sink to a source of warming-inducing gases.

The results presented in Table 1 and Fig. 2 show the relationship among vegetation cover (as proxy from plant greenness), basal peat age and base flux of methane. Although the connection between vegetation composition and cover has been well documented before^15^, the combination with very young peat age data is new. Our data suggest that the oldest parts of the fen peatland complex are not only closer to the flow lines but also associated with higher fluxes despite similar vegetation cover. Similarly, the results indicate the highest mcrA gene abundance in the two oldest, deepest and lowest SOC basal peat samples (Table S1). We interpret this variability in methanogenic abundance as follows: under more anaerobic conditions, methanogenesis dominates despite the low energy yield for the microorganisms, while more energy-efficient anaerobic processes dominate in shallow, carbon-rich samples, lowering the methanogen abundance^24^.

An analysis of the flux emission magnitude as a function of the age of peatland development shows a rapid increase in methane emissions towards a base level obtained after 100 years (Fig. 2). By further (1) combining our data with the range of contemporary observed CO_2_ balance from the same ecosystem^7^ and (2) applying a standard global-warming potential (GWP) conversion of CH_4_ emissions to CO_2_-equivalents^25^, our analysis reveals that the GHG balance likely shifts from an initial sink to a source of C as the methane emissions strengthen after less than 100 yrs. The reverse shift (from source to sink) is well documented for subarctic fen peatland ecosystems over timescales from 1000 to 9000 years e.g. ^26^. Here, we find an initial sink-to-source shift that may take place at a much faster pace and within a timeframe covered by present-day climate projections from, e.g., the state-of-the-art CMIP6 Earth System Models^27,28^ (regardless of the SSP emission scenario) at a 100–300-year timescale. Consequently, the positive feedback mechanism shown here at the centennial scale (which is shaped by the combined climate and peatland development) will most likely be replaced over a the millennial timescale by a gradual cooling effect as the peatlands develop and accumulate greater amounts of peat^26,29^. On the shorter centennial time scale, it should also be kept in mind that from a climate feedback perspective, the natural ecosystem emission dynamics need to be considered in light of the current and future impacts of anthropogenic emissions^30^.

This study has clear limitations in the number of soil samples obtained versus available flux data locations. However, assuming the documented process will be applicable to widespread greening and wetting of natural high Arctic tundra areas, this rapid conversion over just 100 years points to a highly dynamic shift in greenhouse forcing from natural ecosystems with possible implications for climate projections in the 100–300-year timeframe. Further detailed studies from Zackenberg and other similar sites with early peat development will be necessary.

## Methods

### Spatial mapping

An unmanned aerial vehicle (UAV) recorded data on 3 August 2020 at a spatial resolution of 5 × 5 cm. In addition, the normalised difference vegetation index (NDVI) map based on multispectral data indicates variability in plant photosynthetic activity, while the natural colour orthophoto serves as an overview of the study area. Maps of the stream order were drawn using ESRI ArcMap 10.8.

### Fen core sampling & physical soil analyses

In 2020, five fen cores were sampled, of which the deepest 2 cm were separated and stored at 4 °C until later laboratory analyses. The weight-based relative soil organic matter and water content were determined by loss on ignition at the Department of Ecoscience, Risø, Aarhus University, and radiocarbon dating was performed at the Department of Geology, Lund University, as described previously^22,31^.

### mcrA quantitative gene abundance

Genomic DNA from the methanogen *Methanosarcina barkeri* DSM 8687 (Leibniz Institute DSMZ—German Collection of Microorganisms and Cell Cultures GmbH, Germany) in the five fen cores was extracted according to the manufacturer’s instructions in triplicate with a DNeasy® Powerlyzer® Microbial Kit (Qiagen, Hilden, Germany) and quantified with a Qubit® 2.0 Fluorometer (Thermo Fisher Scientific, Life Technologies, Roskilde, Denmark). Simultaneously, the mcrA (methyl-coenzyme M reductase) gene copy numbers were determined by counting cells (100 × 1.30 plan-neofluar Zeiss oil immersion) in an anaerobic *Methanosarcina barkeri* culture after cells of known optical density were fixated in 4% formaldehyde solution and stained with 1:10 diluted Acridine Orange. With the *M. barkeri* DNA extracts as standards, the samples’ mcrA gene abundance was measured with qPCR utilising 10 µM primers mlas (5’-GGTGGTGTMGGDTTCACMCARTA-3’) and mcrA-rev (5’-CGTTCATBGCGTAGTTVGGRTAGT-3’)^16^, 5 × HOT FIREPol® EvaGreen® qPCR Supermix Polymerase (Solis Biodyne, TAG Copenhagen A/S, Copenhagen, Denmark) at 95 °C for 12 min., followed by 35 cycles of 15 s at 95 °C, 30 s at 55 °C and 30 s at 72 °C.

### Methane flux studies

The data presented in the top two rows (F1 and F2) of Table 1b report mean values measured from automatic chambers between July and August in each year during the 2006–2019 period. F1 represents the inner 6 chambers, and F2 represents the outer 4 (Mastepanov et al., in prep.). The remaining studies (F3-6) used unmanipulated chamber measurements during June–August in different years. All studies used the closed-chamber technique, although with different setups (i.e., gas analysis, chamber design, sample time, plot number, and measurement frequency; see Table 2 in Scheller et al.^4^).

### Net ecosystem exchange (NEE) of CO_2_

The available CO_2_ data from the Zackenberg fen consist of high temporal resolution measurements between 2008 and 2018 using the eddy covariance (EC) technique^7^. The EC system was equipped with a closed-path infrared gas analyser LI-6262 and 3-D sonic anemometer Gill R2 until August 2012, when it was upgraded to an enclosed-path LI-7200 and Gill HS. The sonic anemometer in Zackenberg was installed at a height of 3 m (the air intake was attached at the same level). High-frequency (10 Hz) CO_2_ concentration and wind component data were processed according to FLUXNET/ICOS community technique standards^7^. The aggregated 30 min NEE fluxes were further quality-checked and postprocessed using a gap-filling technique based on marginal distribution sampling from the ReddyProc R tool^32^. Detailed information on the EC system setup, flux preprocessing, quality checks of the systems, and data postprocessing (NEE gap-filling) can be found in reference ^7^. The CO_2_ fluxes reported in Table S2 follow the standard micrometeorological sign of convention, which is CO_2_ uptake and release as negative and positive fluxes, respectively.

## Supplementary Information

Supplementary Tables.

## Data Availability

The long-term flux datasets analysed and used during the current study are available in the open-source repository Greenland Ecosystem Monitoring Database https://data.g-e-m.dk. The radiocarbon and molecular datasets are available from the corresponding author upon reasonable request.
