# Supplementary material for: Rapid shift in greenhouse forcing of emerging arctic peatlands
Source: Sci Rep. 2023 Feb 17;13:2828. doi: 10.1038/s41598-023-29859-4 (PMC9938109; doi:10.1038/s41598-023-29859-4)
Supplement: Supplementary file 1 — Supplementary Tables. [file 41598_2023_29859_MOESM1_ESM.docx]

*Table S1: Positions and characteristics of cores taken for Basal Peat (BP) analysis. The different BP depths reflect the extend of organic layer development on a minerogenic silt base in the respective places. In italics repeat information from Table 1.*

| Sample | C1 | C2 | C3 | C4 | C5 |
| --- | --- | --- | --- | --- | --- |
| Coordinates | 74.4822, -  20.5562 | 74.4822, -20.5576 | 74.4807, -20.5567 | 74.4790, -20.5559 | 74.4786, -20.5564 |
| BP core | 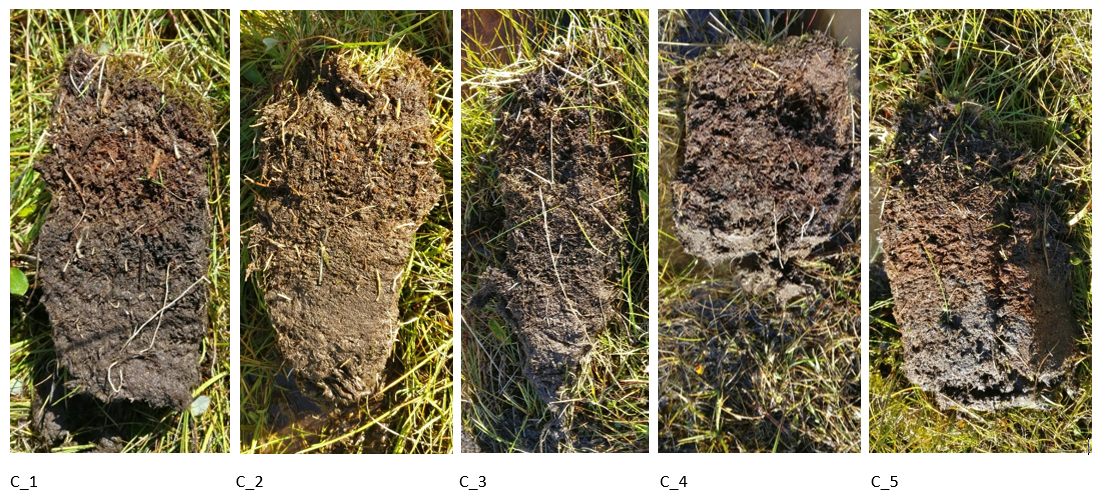 | 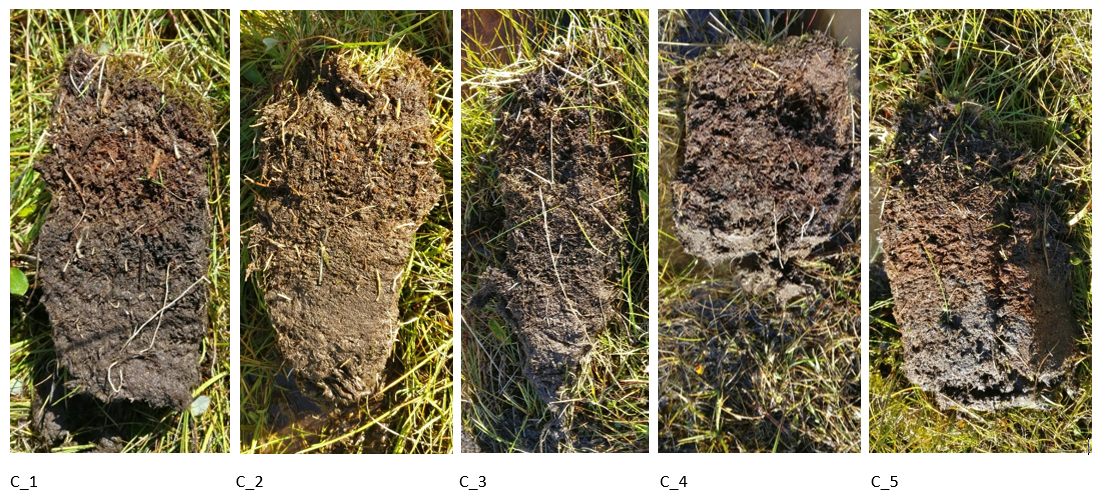 | 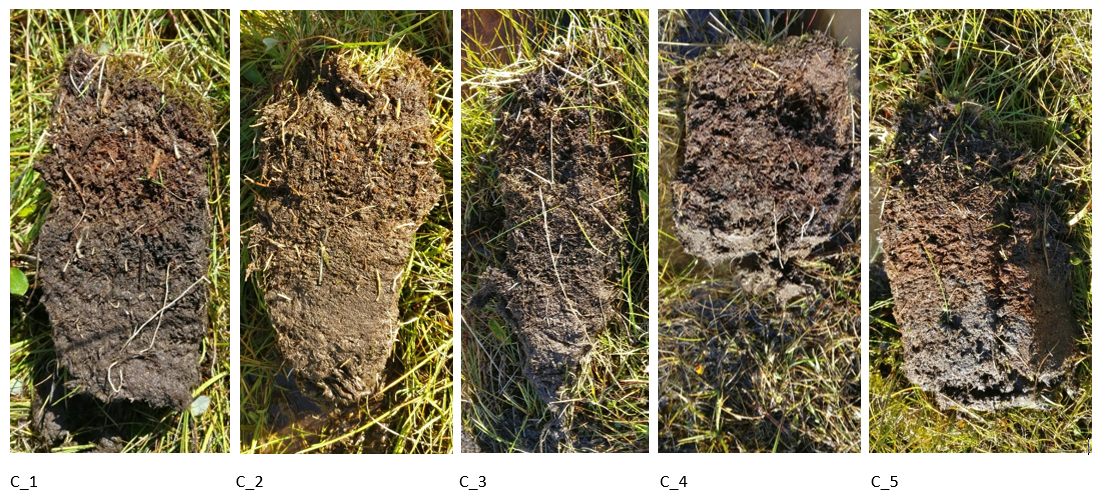 | 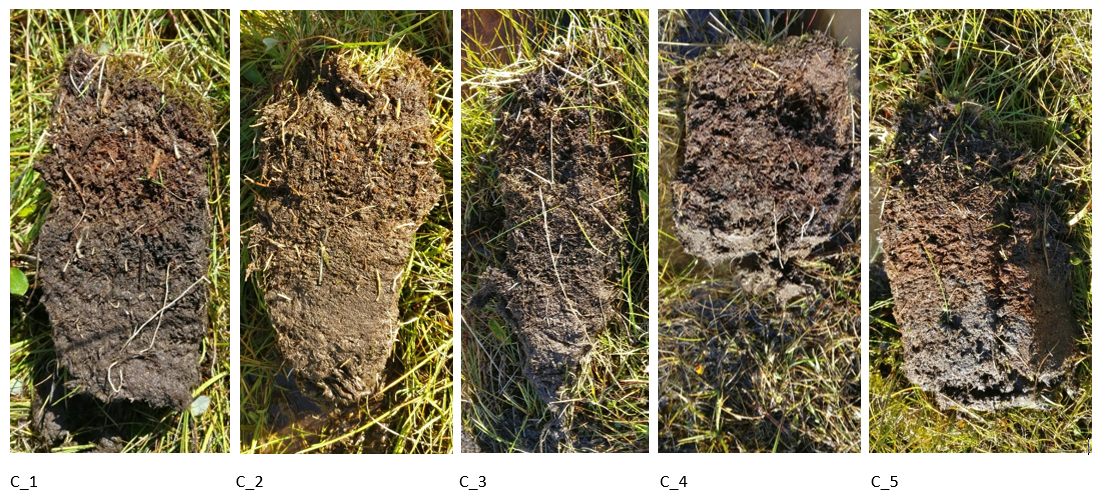 | 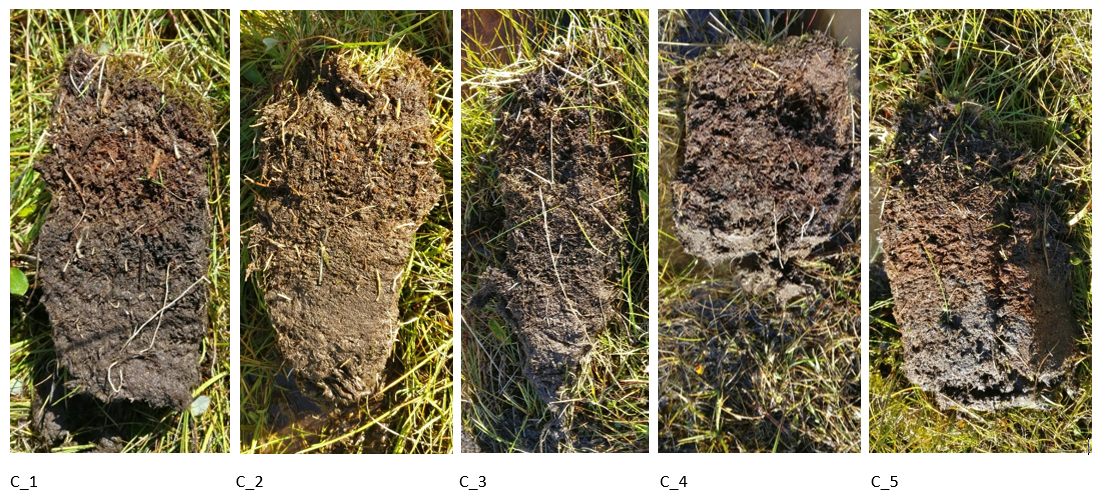 |
| BP depth (cm) | 30 | 30 | 45 | 15 | 30 |
| BP organic matter (%) | 49.7 | 47.5 | 40.0 | 51.1 | 33.9 |
| mcrA concentration [fg/µl] | 3.80 ± 1.87 | 4.05 ± 1.21 | 19.74 ± 4.61 | 0.96 ± 0.12 | 7.75 ± 3.54 |
| 14C age BP | 100 ± 30 | 1.492 ± 0.0006 fM | 180 ± 30 | 60 ± 35 | 210 ± 30 |

*Table S2: Interannual variability in current net CO_2_ uptake of the arctic fen ecosystem as obtained through eddy covariance measurements. The interannual range shown below characterizes the CO_2_ uptake used in Figure 2 as varying between an C uptake of -90 g CO_2_ m^-2^ yr^-1^ and release of +21 g CO_2_ m^-2^ yr^-1^. These annual estimates for uptake and net balance represent a maximum as winter losses are not fully covered in the measurements (only March-November period are reported). Additionally, years with more than half growing season missing data (i.e. 2009, 2010, and 2012) have been omitted in this calculation.*

| Year | NEE (g CO_2_ m^-2^ yr^-1^) | NEE uncertainty (g CO_2_ m^-2^ yr^-1^) |
| --- | --- | --- |
| 2008 | -64.9 | 1.1 |
| 2011 | -75.8 | 1.2 |
| 2013 | -49.1 | 1.0 |
| 2014 | -48.5 | 1.0 |
| 2015 | -29.7 | 1.0 |
| 2016 | -90.0 | 1.0 |
| 2017 | -62.8 | 0.8 |
| 2018 | 21.4 | 1.0 |
| 2008-2018 | -49.9 | 1.0 |
